# One Year Into the Pandemic: A Systematic Review of Perinatal Mental Health Outcomes During COVID-19

**DOI:** 10.3389/fpsyt.2021.674194

**Published:** 2021-06-24

**Authors:** Udita Iyengar, Bhavisha Jaiprakash, Hanako Haitsuka, Sohye Kim

**Affiliations:** ^1^Department of Child and Adolescent Psychiatry, King's College London, Institute of Psychiatry, Psychology, and Neuroscience (IoPPN), London, United Kingdom; ^2^Yale Child Study Center, Yale University, New Haven, CT, United States; ^3^Eunice Kennedy Shriver Center, University of Massachusetts Medical School, Worcester, MA, United States; ^4^Departments of Psychiatry, Pediatrics, and Obstetrics and Gynecology, University of Massachusetts Medical School, Worcester, MA, United States

**Keywords:** pregnancy, postpartum, mental health, COVID-19, maternal, perinatal

## Abstract

Obstetric guidelines have rapidly evolved to incorporate new data and research on the novel coronavirus disease (COVID-19), with data on perinatal mental health building over the last year. Our aim in the present manuscript is to provide a systematic review of mental health outcomes in pregnant and postpartum women during the COVID-19 pandemic in the context of neonatal and obstetric guidelines addressing symptoms and complications of COVID-19 during pregnancy, mother-to-neonate transmission, Cesarean-section delivery, neonatal prematurity, maternal/neonate mortalities, maternal-neonatal separation, and breastfeeding. We summarize data from 81 mental health studies of pregnant and postpartum women and underscore protective and risk factors identified for perinatal mental health outcomes amidst the COVID-19 pandemic. Data reviewed here suggest increased psychological symptoms, especially depressive and anxiety symptoms, in pregnant and postpartum women during COVID-19. Our systematic review integrates the most current obstetric and neonate guidelines, along with perinatal mental health outcomes associated with COVID-19, highlighting the best available data for the care of women and their neonates amidst the current COVID-19 pandemic.

## Introduction

Since being declared a pandemic in March 2020 by the World Health Organization (WHO), the novel coronavirus disease (COVID-19) has rapidly spread across the globe (1). As of April 12th 2021, there have been nearly 139 million confirmed cases and nearly 3 million deaths worldwide (2, 3). These staggering figures have resulted in an array of public health, social, and economic issues impacting the daily life and mental health of the global population. Over the last year of the COVID-19 pandemic, there have been growing reports of the mental health impacts of COVID-19 on the general population, including increased depression, anxiety, and sleep disturbances in individuals with and without COVID-19 (4–7).

Even under normal circumstances, the perinatal period is already one of substantial biological, physiological, psychological, and social changes. For example, pregnant women may be more susceptible to and more significantly impacted by viral diseases, as seen in the H1N1 influenza pandemic [swine flu in 2009; (8, 9)], SARS-CoV [Severe Acute Respiratory Syndrome identified in 2003; (10)] and MERS-CoV [Middle East Respiratory Syndrome identified in 2012; (11)]. In addition, pregnant women tend to be more susceptible to severe symptoms if contracting a respiratory viral illness, increasing the risk of adverse obstetric outcomes such as preeclampsia (12), preterm delivery (13), and low birth weight birth (14). The backdrop of a global pandemic is likely to exacerbate any inherent risks of contracting a respiratory viral illness during the perinatal period (15–18). Therefore, pregnant women and their newborns have already been proposed as a special vulnerable group requiring our priority and attention amidst the current COVID-19 outbreak (15–20).

### Mental Health During Pregnancy and Postpartum Period

Mental health disorders are not uncommon during pregnancy and the postpartum period, and studies estimate that the prevalence rate of perinatal mood and anxiety disorders range between 10 and 20% (21–24). In addition to universal challenges experienced during the global pandemic, pregnant women are likely to be affected by a unique set of additional challenges, such as limited access to perinatal services and in-person family support. In addition, constantly evolving understanding of the potential risks posed by the novel virus may contribute to a heightened sense of uncertainty about their own health and the health of their newborn, which may intensify the level of stress experienced during this critical time of transition. One year into the pandemic, a growing body of perinatal mental health studies have emerged, and with the onset of new virus variants as well as the development of vaccines, there is bound to be a greater influx of studies capturing the mental health experience of women during pregnancy and the postpartum period.

### Summary of Obstetric and Neonatal Outcomes During COVID-19

Considering that growing and fluctuating concerns about adverse obstetric and neonatal outcomes may have been inextricably tied to increased perinatal mental health risk during COVID-19, we review perinatal mental health outcomes in the context of key obstetric and neonatal outcomes that have been documented to date. In this section, we draw from comprehensive published guidelines detailing obstetric and neonatal outcomes for practitioners and the general public from leading sources (25–29) and summarize data concerning symptoms and complications of maternal COVID-19 during pregnancy, mother-to-neonate transmission, Cesarean-section delivery, premature birth, maternal/neonatal mortalities, maternal-neonatal separation, and breastfeeding during COVID-19. Our summary of obstetric and neonatal data is not intended to be exhaustive but is rather aimed at providing the context in which to understand current perinatal mental health outcomes.

#### Symptoms and Complications of COVID-19 During Pregnancy

While most pregnant women infected with COVID-19 were asymptomatic (25), fever, mild cough, and dyspnea were the most reported symptoms of COVID-19 during pregnancy. Most frequent pregnancy complications included gestational diabetes, preeclampsia, and premature rupture of membranes. According to an interim report from the United Kingdom (UK) Obstetric Surveillance System (UKOSS) national cohort study comparing pregnant and non-pregnant women with COVID-19, pregnant women were more likely than their non-pregnant counterparts to be hospitalized, to be admitted more frequently to the intensive care unit (ICU) and to require greater use of mechanical ventilation (30). Maternal race and ethnicity have received attention in relation to risks and complications associated with COVID-19. In UK, women hospitalized with symptomatic COVID-19 infections were more likely to be Black, Asian, or another ethnic minority (25). Non-Hispanic Asians reported more frequent ICU admissions compared to all pregnant women. Cohort studies in the United States (US) also reported disproportionate risk based on race and ethnicity, with increased risk for ICU admission noted among non-Hispanic Asian pregnant women and non-Hispanic Native Hawaiian/Pacific Islander pregnant women (31). In addition, a significantly higher infection rate was observed among Hispanic pregnant women compared with non-Hispanic White pregnant women (18.1 vs. 9.4%) (32). Age was also a significant factor, with women aged 35–44 reporting more frequent hospitalization, ICU admission, mechanical ventilation, and death compared to women aged 15–24 (30).

#### Mode of Delivery

Data from the US Center for Disease Control and Prevention (CDC) suggested that of 11,764 deliveries that took place over a period from March 29th, 2020 to February 10th, 2021, 7,279 were vaginal while 3,492 were C-sections (30%) (33). The C-section rates during this period is roughly similar to averge rates (31%) reported in pre-pandemic years (34). It is advised that the mother with suspected or confirmed COVID-19 deliver in an obstetric unit and a discussion weighing the benefits and costs of elective C-section or induction of labor take place should a pregnant mother test positive for COVID-19 (25). This aligns with WHO's recommendation that C-sections only be done when medically justified (35). Elective C-sections of women with suspected or confirmed COVID-19 should remain the lowest priority on the operating list (25). Furthermore, if mother is COVID-19 positive and an emergency C-section is required, the mother and her family are advised to wear proper personal protection equipment (25) during delivery and while at the hospital.

#### Neonatal Prematurity

Guidelines have reported on the prevalence of premature birth during the COVID-19 pandemic and addressed the issue of neonatal intensive care unit (NICU) admission. Pregnant women testing positive for COVID-19 may be at an increased risk for preterm birth (29). The risk rises in those women who are symptomatic (25), and furthermore in those with severe, compared to mild, COVID-19 symptoms (36). UK surveillance data indicates that preterm births in women with symptomatic COVID-19 are to be two to three times higher (30) than the pre-pandemic preterm birth rate of 7% in the UK (37). However, recent surveillance data from the US reported 1,131 preterm births (compared to 8,331 term births) between March 29th, 2020 and February 10th, 2021 (33), consistent with the pre-pandemic global preterm prevalence rate of 10% (38). If mothers pose a risk of transmitting COVID-19 to their preterm infant, it is currently understood that it may be necessary to separate mother and her newborn (39).

#### Mother to Fetus/Neonate Transmission

A major concern since the start of the pandemic has centered around whether the virus can be transmitted from mothers with COVID-19 to the fetus/neonate during pregnancy or delivery. The current advice suggests that infants born to mothers with confirmed COVID-19 be treated as having been potentially infected (39), and that certain precautions be taken if mothers are currently positive for COVID-19 (e.g., temporary separation from infants and the use of a breast pump to avoid the spread of droplets while being in close contact with their infant). Regarding breastfeeding, long-term benefits are understood to outweigh the potential risk of transmission (25). To date, active virus has not been found in samples of breastmilk (35). Furthermore, surveillance data from the US found that only 60 of 1,506 infants of mothers with confirmed COVID-19 tested positive for COVID-19, while 1,436 infants tested negative (33), suggesting low risk of mother-to-neonate transmission. Two studies have further reported no evidence of vertical transmission (36, 40), while one study found placental abnormalities in mothers with COVID-19 (41). In sum, the currently available data suggests that the rate of infection of COVID-19 from mother to infant is low, and studies are currently unable to conclusively determine if the neonate's positive COVID-19 status has been due to vertical transmission, or if the virus was contracted post-delivery.

#### Maternal and Fetal/Neonatal Mortalities

Currently available data report a low rate of deaths among pregnant mothers with COVID-19; however, the risk of death increased if women had symptomatic COVID-19 requiring hospitalization (25). Two studies reported on causes of maternal death, including pre-existing conditions, obstetric complications, and respiratory complications due to COVID-19 (42, 43). The UKOSS study reported no difference between pregnant and non-pregnant women in the risk of death due to COVID-19 (30). In the US, Black and Hispanic pregnant women had disproportionate rates of death associated with COVID-19 infection compared to other racial and ethnic groups (39).

#### Mother-Neonate Separation and Breastfeeding

As a result of the rapid spread of COVID-19 and the limited understanding of the specific mechanism of transmission at the start of the pandemic, interim guidelines by the American Academy of Pediatrics (AAP) and CDC recommended temporary separation of mother and neonate following delivery, potentially disrupting skin-to-skin and breastfeeding practices (44, 45). Some sounded the alarm for an urgent need for perinatal mental health specialists in the NICU during and after the pandemic (46), due to the additional stress that the separation and NICU admission can place on mothers and infants (46–48). Updated guidelines proposed by WHO (28) and RCOG (25) now advise against routine separation and encourage mother-to-infant contact, though shared decision making between the mother and the medical team is crucial and should be reviewed on a case-by-case basis (25, 29).

Concerns about compromised breastfeeding practices during the pandemic have been raised (49–51). Recommendations early in the pandemic advised against mothers with suspected/confirmed COVID-19 to breastfeed, unless their breastmilk tested negative for COVID-19 (52). Recent guidelines support breastfeeding in mothers with and without COVID-19, suggesting that benefits of breastfeeding outweigh any potential risks of transmission through breastmilk. Guidelines provide recommendations on minimizing the risk of transmission during breastfeeding, such as washing hands thoroughly before feeding or expressing milk, wearing a face covering while feeding the infant, or having a healthy family member feed the infant (25, 29).

### Current Study

Our aim in the present study is to provide a systematic review of mental health outcomes in pregnant and postpartum women during the COVID-19 pandemic in the context of key neonatal and obstetrics guidelines as summarized above. Much of the available data on perinatal mental health outcomes have focused on depression and anxiety symptoms and we review this data with a special attention to protective and risk factors.

## Method

### Identification and Selection of Studies

We selected published studies that had a primary focus on the impact of COVID-19 and measured a specific mental health outcome using validated measures. We included studies with a primary population of pregnant women (at any stage of pregnancy) and women up to 1.5 years postpartum. We excluded studies not written in English, case studies or case reports of < 10 participants, guidelines or proposals of patient care and management, or studies that did not measure a mental health outcome using a validated measure.

### Literature Search Strategy

A literature search was conducted on January 31st, 2021. Selected search engines included Embase, Medline and PsycInfo. Search strategies included keywords pertaining to the COVID-19 pandemic (novel corona, SARS 2, SARS COV-2, corona, coronavirus). These terms were combined with terms relating to pregnancy (perinatal, neonatal, intrapartum, postpartum, postnatal, mother) and terms related to mental health (depression, anxiety, mental health, well-being). Complete search strategies for each database are provided in Appendix 1 of Supplementary Material.

### Data Extraction

**Figure 1:**
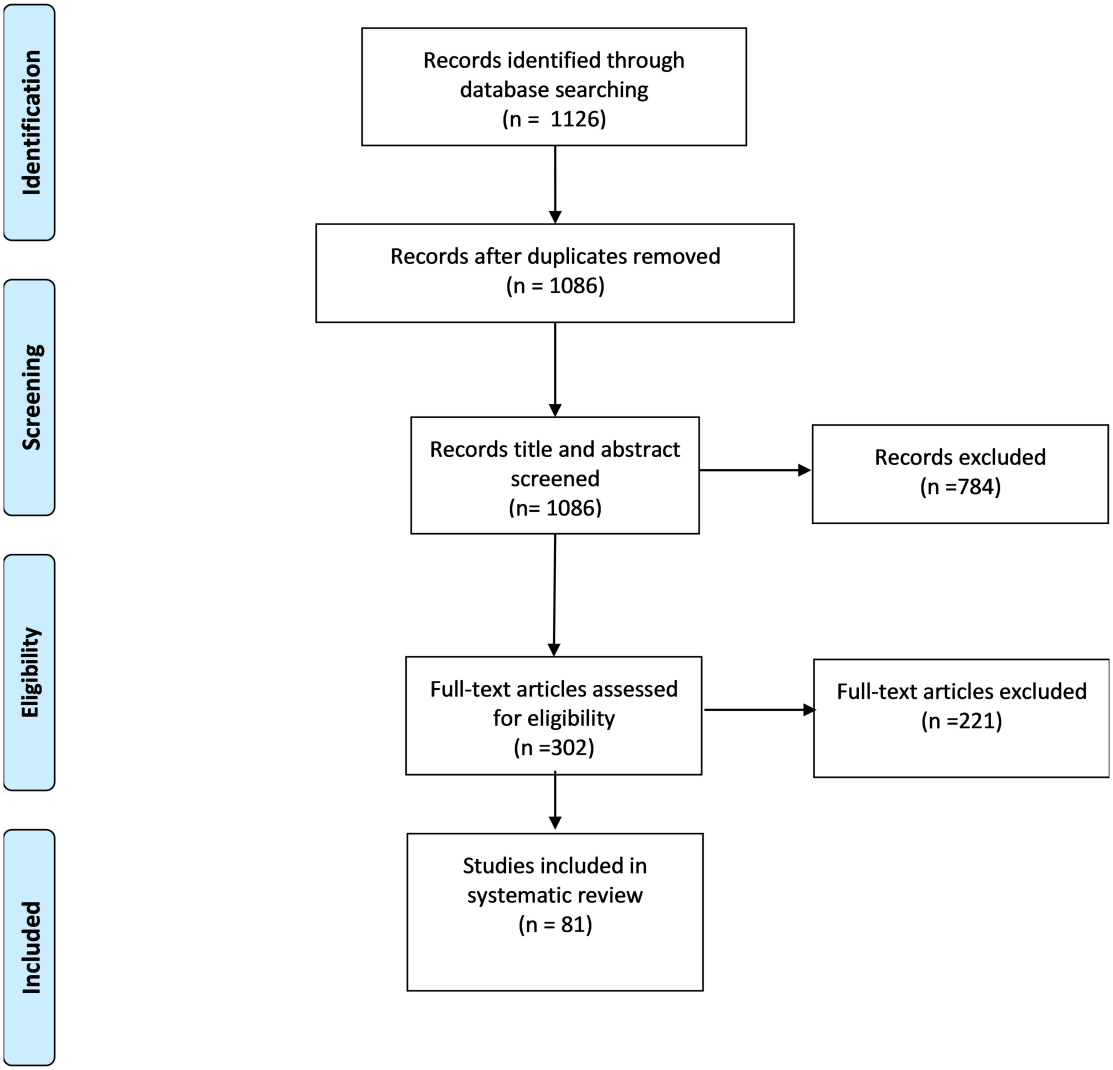
PRISMA flow diagram of the systematic search (53).

After duplicates were removed, articles underwent title and abstract screening (BJ, UI, HH). All selected full-text articles were then individually read and designated information was retrieved (BJ, UI, HH). Results from the screening process are presented in a PRISMA flow diagram (Figure 1) (53). A total of 1,126 articles were identified from the search engines. Following an initial title and abstract screening of 1,086 articles and excluding 784 records, 302 full-text articles were retrieved and assessed for eligibility, and 221 were excluded further, resulting in 81 articles reporting perinatal mental health outcomes during COVID-19.

A coding protocol for all articles was used to extract and categorize the following information: (1) title, authors, and year of publication; (2) study characteristics (country of origin, study period, recruitment method, participant characteristics, sample size, study design); (3) comparison group (e.g., before vs. during pandemic, within-group comparisons); and (4) primary mental health outcomes and risk/protective factors.

### Assessment of Risk of Bias

To evaluate risk of bias, we used a modified version of the Newcastle-Ottawa Scale [adapted for cross-sectional studies; (54)], which evaluates studies in terms of selection of groups, comparability of groups, and outcome assessment. Scores range from 0 (highest bias) to 9 points (lowest bias). Before performing quality assessment, the criteria for evaluation were thoroughly discussed between three raters (UI, BJ, HH) to develop a common understanding. Ratings for each study was determined by two raters (BJ and HH), and any uncertainties concerning study quality were resolved through discussions between all raters (BJ, HH, UI).

## Results

### Study Selection

A total of 81 studies met our criteria outlined above. Of the 81 studies (*N* = 132,917 women), 55 studies included pregnant women only (*n* = 95,353), 13 studies included both pregnant and postpartum (up to 1.5 years) women (*n* = 25,834), and 13 studies included postpartum women only (*n* = 11,730). Study characteristics and main findings of all studies are summarized in Tables 1–9.

**Table 1:** Prevalence of mental health outcomes in pregnant or postpartum women during the COVID-19 pandemic.

**Study**	**Study Design**	**Country**	**Study Period**	**Recruitment Site/Method**	**Participant Characteristics**		**Main Findings**	**Risk of Bias^a^**
					**Pregnancy/ Postpartum Status**	**Maternal Age**		
Ceulemans et al. (55)	Cross-sectional	Belgium	Lockdown period (during the pandemic)	Hospitals	Pregnant (*n =* 2,421) and postpartum (*n =* 3,445) women (*N =* 5,866)	NR	25.3% and 23.6% of participating pregnant and postpartum women reported depressive symptoms. 14% of all participating women met criteria for high anxiety.	3
Effati-Daryani et al. (56)	Cross-sectional	Iran	March–April 2020 (during the pandemic)	Health centers	Pregnant women (*N =* 205) • <14 weeks GA: *n =* 16 • 14–28 weeks GA: *n =* 85 • >28 weeks GA: *n =* 104	29.5 (M) ± 5.5 (SD) years	32.7% of participating women experienced depressive symptoms and 43.9% experienced anxiety symptoms, comparable to pre-pandemic prevalence rates. *Protective factors (for anxiety):* marital life satisfaction, high level of spousal education, and high income	7
Farewell et al. (57)	Cross-sectional	United States of America	March–April 2020 (during the pandemic)	Social media	Pregnant (*n =* 14) and postpartum (<6 months; *n =* 13) women (*N =* 27)	(R) 24–34 years: *n =* 15 35–45 years: *n =* 12	12% of participating women reported high depressive symptoms and 60% reported moderate to severe anxiety. *Risk factors:* high uncertainty around prenatal care appointment, prenatal exposure risk, social isolation, stress about lack of daycare and caregiver support	6
Farrell et al. (58)	Cross-sectional	Qatar	June–July 2020 (during the pandemic)	Maternity hospital	Pregnant women (*N =* 288) • GA: 26.1 (M) ± 14.3 (SD) weeks	30.5 (M) ± 5.3 (SD) years	39.2% of participating women experienced depression and 34.4% experienced anxiety.	6
He et al. (59)	Cross-sectional	China	February 13–16, 2020 (during the pandemic)	Maternity school	Postpartum women (*N =* 1,908)	NR	58% of participating women screened positive for postpartum depression and 15% screened positive for PTSD. *Risk factors*: low levels of education, fear of infection	5
Hocaoglu et al. (60)	Cross-sectional	Turkey	May 11–28, 2020 (during the pandemic)	Prenatal checks	Pregnant women (*N =* 283) • GA: 23.82 (M) ± 11.05 (SD) weeks	29.2 (M) ± 5.55 (SD) years	High rates of anxiety and PTSD were reported among participating women, with 46.6% reporting severe impact during the COVID-19 pandemic. *Risk factors (for anxiety):* pregnancy complications and husband's employment status; *(for PTSD):* presence of COVID-19-related symptoms and high education level	8
Kassaw et al. (61)	Cross-sectional	Ethiopia	April 6–May 6, 2020 (during the pandemic)	Hospitals	Pregnant women (*N =* 178)	28 (M) ± 5.6 (SD) years	1/3 of participating women had generalized anxiety disorder. *Risk factors:* rural area resident status, high level of education, poor social support, and primigravida.	7
Liang et al. (62)	Cross-sectional	China	March 30–April 13, 2020 (during the pandemic)	Hospitals	Postpartum (6–12 weeks) women (*N =* 864)	(R) 25–29 years: *n =* 355	30% of participating women experienced depression. *Risk factors:* immigrant status, persistent fever, poor social support, concerns about COVID-19 infection	6
Liu et al. (17)	Cross-sectional	United States of America	May 21–August 17, 2020 (during the pandemic)	Social media	Pregnant and postpartum ( ≤ 6 months) women (*N =* 1,123) • 2nd trimester: *n =* 441 • 3rd trimester: *n =* 682	33.1 (M) ± 3.77 (SD) years	36.4% of participating women reported depression, 22.7% reported generalized anxiety and 10.3% reported PTSD symptoms. *Risk factors*: previous psychiatric history, COVID-19 health worries and grief	9
Lubian-Lopez et al. (63)	Cross-sectional	Spain	April 15–May 14, 2020 (during the pandemic)	Prenatal clinics	Pregnant women (*N =* 454) • GA: 26.10 (M) ± 8.7 (SD) weeks	32.5 (M) ± 4.53 (SD) years	35.9% of participating women showed depressive symptoms and 45.6% had anxiety symptoms.	6
Medina-Jiminez et al. (64)	Population-based	United States of America	May 5–June 12, 2020 (during the pandemic)	Hospitals	Pregnant women (*N =* 478) • GA: 27.9 (M) ± 10.3 (SD) weeks	28.1 (M) ± 6.25 (SD) years	33.2% of participating women reported high stress and 17.5% reported high levels of depression. *Risk factors*: later gestational age.	7
Molgora & Accordini (65)	Cross-sectional	Italy	March 1–May 3, 2020 (during the pandemic)	Social media	Pregnant (*n =* 389) and postpartum women (<6 months; *n =* 186) (*N =* 575)	Pregnant: 32.9 (M) ± 4.3 (SD) years Postpartum: 33.01 (M) ± 4.19 (SD) years	60% of pregnant and 57.7% of postpartum women reported clinically significant state anxiety. 34.2% of pregnant and 26.3% of postpartum women reported clinically significant depression. 16.7% of postpartum women reported PTSD. *Risk factors:* lack of presence and social support from partner during delivery and early postpartum	7
Ng et al. (66)	Cross-sectional	Singapore	March 31–April 25, 2020 (during the pandemic)	Prenatal clinics and hospitals	Pregnant women (*N =* 324) • GA: 23.4 (M) ± 10 (SD) weeks	31.8 (M) ± 4.2 (SD) years	35.8% of participating women screened positive for anxiety and 18.2% for depression. *Risk factor (for anxiety):* cognitive association of COVID-19 with fetal anomalies/death	7
Ostacoli et al. (67)	Cross-sectional	Italy	March 8–June 15, 2020 (during the pandemic)	Hospitals	Postpartum women (*N =* 163)	34.77 (M) ± 5.01 (SD) years	44.2% of participating women reported depressive symptoms and 42.9% reported PTSD symptoms. *Risk factors:* dismissive and fearful avoidant attachment, and perceived pain during birth. *Protective factors:* perceived support by healthcare staff and quietness due to lack of visitors	7
Pries et al. (68)	Cross-sectional	United States of America	April 2020 (during the pandemic)	Social media	Pregnant women (*N =* 788) • GA: 25.3 (M) ± 9.1 (SD) weeks	29.2 (M) ± 5.3 (SD) years	21.1% of participating women reported no to minimal anxiety, 35.6% reported mild anxiety, 21.6% reported moderate anxiety and 21.7% reported severe anxiety. *Risk factors*: previous abuse history, high-risk pregnancy, and perinatal infection stress. *Protective factors:* older maternal age and better prenatal health	6
Ravaldi and Vannacci, (69)	Cross-sectional	Italy	March–May 2020 (during the lockdown phase of the pandemic)	Social media	Pregnant women (*n =* 1,307) and postpartum (*n =* 1,141) (*N =* 2,448)	(R) Pregnant women: 18–25 years: *n =* 18 25–30 years: *n =* 187 30–35 years: *n =* 497 >35 years: *n =* 605 Postpartum women: 18–25 years: *n =* 21 25–30 years: *n =* 131 30–35 years: *n =* 465 >35 years: *n =* 524	45.7% of pregnant and postpartum women had personal experience of psychopathology, and 46.9% had family history of psychopathology	5
Yang et al. (70)	Cross-sectional	China	February 25–March 10, 2020 (during the pandemic)	Hospitals	Pregnant women (*N =* 19,515) • 1–10 weeks GA: *n =* 1,523 • 11–20 weeks GA: *n =* 4,986 • 21–30 weeks GA: *n =* 5,858 • >30 weeks GA: *n =* 6,518	(R) <26–35 years: *n =* 3,781 26–30 years: *n =* 8,202 31–35 years: *n =* 5,683 >35 years: *n =* 1,849	44.6% of participating women reported depressive symptoms, 29.2% reported anxiety symptoms and 7.4% had suicidal ideations. *Risk factor*: perceived low social support	
Yue et al. (71)	Cross-sectional	China	February 2020 (during the pandemic)	Online	Pregnant women (*N =* 308) • GA: 31.63 (M) ± 2.22 (SD) weeks	31.02 (M) ± 3.91 (SD) years	Anxiety in pregnant women during the pandemic was higher than that of the general population prior to the COVID-19 pandemic, including the pregnant and non-pregnant population.	6

*GA, gestational age; M, mean; SD, standard deviation; Med, median; R, range; PTSD, post-traumatic stress disorder; NR, not reported*.

a *Assessed using a modified version of the Newcastle-Ottawa Scale (54). See section Assessment of Risk of Bias for details. Scores range from 0 (highest bias) to 10 points (lowest bias)*.

**Table 2:** Mental health outcomes in pregnant or postpartum women before vs. during the COVID-19 pandemic.

**Study**	**Study Design**	**Country**	**Recruitment Sites/Methods**	**Participant Characteristics**			**Main Findings**	**Risk of Bias^b^**
				**Subgroups**	**Pregnancy/ Postpartum Status**	**Maternal Age**		
Ayaz et al. (72)	Cross-sectional	Turkey	Outpatient prenatal clinic	**Pandemic** (April 12–May 27, 2020; *n =* 63) vs. **Pre-pandemic** (June 2018-; end date and *n*: NR)	Pregnant women (*N =* 63) • GA: 32.5 (M) ± 7 (SD) weeks	30.4 (M) ± 5.3 (SD) years	Pregnant women reported more depressive and anxiety symptoms during compared to before the pandemic. *Risk factors:* obesity, negative relationship with husband	8
Berthelot et al. (73)	Case-control/ Longitudinal	Canada	Social media and prenatal clinics	**Pandemic** (April 2020; *n =* 1,258) vs. **Pre-pandemic** (April 2018–March 2020; *n =* 496)	Pregnant women (*N =* 1,754) • GA: [pandemic] 24.38 (M) ± 9.2 (SD) weeks; [pre-pandemic] 25.8 (M) ± 9.73 (SD) weeks	29.27 (M) ± 4.23 (SD) years	Pregnant women reported more severe symptoms of depression, anxiety and PTSD during compared to before the pandemic.	7
Cameron et al. (74)	Cross-sectional	Canada	Social media	**Pandemic** (April 14–28, 2020; *n =* 312) vs. **Pre-pandemic** (specific dates: NR; *n =* 312)	Postpartum women ( ≤ 0–18 months^a^; *N =* 312)	34.28 (M) ± 5.02 (SD) years	34.09% of postpartum women reported depressive symptoms and 34.55% reported anxiety symptoms during the pandemic. Postpartum women reported increased depressive and anxiety symptoms during compared to before the pandemic.	5
Davenport et al. (75)	Cross-sectional	Canada	Social media	**Pandemic** (April 14–May 8, 2020; *n =* 900) vs. **Pre-pandemic** (retrospective recall; *n =* 900)	Pregnant (*N =* 520) and postpartum (<1 year; *n =* 380) women (*N =* 900)	Med = 33 (R = 17–49) years	15% and 40.7% of participating women met criteria for depression before and during the pandemic, respectively. 39% and 72% of women met criteria for moderate to high anxiety before and during the pandemic, respectively. *Protective factor:* (for depression and anxiety) 150+ mins of physical activity	8
Hui et al. (76)	Retrospective	Hong Kong (People's Republic of China)	Hospital	**Pandemic** (January 5, 2020–April 30, 2020; *n =* 954) vs. **Pre-pandemic** (January 1, 2019–January 4, 2020; *n =* 3,577)	Postpartum women (*N =* 4,531) • GA: [pandemic] 38.5 (M) ± 2.29 (SD) weeks; [pre-pandemic] 38.5 (M) ± 2.25 (SD) weeks	[Pandemic]: 33.1 (M) ± 4.6 (SD) years [Pre-pandemic]: 33.1 (M) ± 4.4 (SD) years	Women who delivered during compared to before the pandemic reported higher depressive symptoms.	6
Loret de Mola et al. (77)	Longitudinal	Brazil	Hospitals	**Pandemic 1st wave** (May–July 2020) vs. **Pandemic 2nd wave** (July–December 2020) vs. **Pre-pandemic** (2019)	Pregnant women (*N =* 591)	NR	In participating pregnant women, depression prevalence rose from a pre-pandemic rate of 3.1% to 28.4% during the first wave in 2020, and to 30.6% during the second wave of the pandemic. Anxiety increased from 9.6% (pre-pandemic) to 26.7% (1st wave), to 28.8% (2nd wave), with a 3-fold increase in prevalence.	4
Matvienko-Sikar et al. (78)	Cross-sectional	Ireland	Social media and hospital	**Pandemic** (June 16–July 17, 2020; *n =* 235) vs. **Pre-pandemic** (May 2019–February 2020; *n =* 210)	Pregnant women (*N =* 445) • GA: [pandemic] 27.49 (M) ± 8.60 (SD) weeks; [pre-pandemic] 26.43 (M) ± 10.09 (SD) weeks	[Pandemic]: 33.67 (M) ± 4.47 (SD) years [Pre-pandemic]: 33.91 (M) ± 4.05 (SD) years	Participating pregnant women reported higher stress during compared to before the pandemic. *Risk factors*: perceived low social support, low physical activity	7
Mayopoulous et al. (79)	Cross-sectional	United States of America	Social media, professional organizations, and hospitals	**Pandemic** (March–April 2020; *n =* 1,611) vs. **Pre- pandemic** (early in 2020 [specific dates NR]; *N =* 637)	Postpartum women (*N =* 1,274)	32.0 (M) years	Postpartum women reported higher acute stress during compared to before the pandemic. Higher acute stress during birth was significantly associated with increased childbirth-related PTSD symptoms and decreased infant bonding.	8
McFarland et al. (80)	Population-based	United States of America	Records for live births	**Pandemic** vs. **Pre- pandemic** (Time matched samples [*N =* 18,531]: September 2019–April 2020; Month-matched samples [*N =* 18,346]: January 2019–April 2019 and January 2020–April 2020)	Pregnant women (*N =* 32,352)	(R) <20 years: *n =* 647 20–34 years: *n =* 22,970–23,617 35+ years: *n =* 7,764–8,735	Pregnant women who gave birth during compared to before the pandemic reported elevated depressive symptoms.	7
Moyer et al. (81)	Cross-sectional	United States of America	Social media	**Pandemic** (April 3–24, 2020; *n =* 2,740) vs. **Pre-pandemic** (retrospective recall; *n =* 2,740)	Pregnant women (*N =* 2,740) • 3rd trimester: *n =* 1,128	M = 32.7 years	Pregnant women reporting more COVID-19 related stressors had the greatest changes in pre- to post- pregnancy-related anxiety. *Risk factors:* lack of face-to-face prenatal visits, change in birth plans away from in-hospital delivery, fear of running out of food, increased conflict at home, fear of infection, essential worker status (self or family member), COVID-19 high-risk area resident status, loss of childcare, loss of job, low education levels, previous mental health disorder	8
Pariente et al. (82)	Cohort	Israel	Hospital	**Pandemic** (March 18–April 29, 2020; *n =* 223) vs. **Pre- pandemic** (November 2016–April 2017; *n =* 123)	Postpartum women (*N =* 346) • GA: [pandemic] 39.4 (M) ± 1.0 (SD) weeks; [pre-pandemic] 39.4 (M) ± 0.9 (SD) weeks	[Pandemic]: 29.1 (M) ± 5.1 (SD) years [Pre-pandemic]: 28.3 (M) ± 5.0 (SD) years	Women delivering during compared to before the pandemic had lower risk of developing postpartum depression.	6
Sade et al. (83)	Cross-sectional	Israel	Hospital	**Pandemic** (March 19–May 26, 2020; *n =* 84) vs. **Pre-pandemic** (November 2016–April 2017; *n =* 279)	Pregnant women in high-risk obstetric units (*N =* 363) • GA: [pandemic] 33.7 (M) ± 5.1 (SD) weeks; pre-pandemic 34.0 (M) ± 4.8 (SD) weeks	[Pandemic]: (R) <20 years: *n =* 2 20–35 years: *n =* 67 >35 years: *n =* 15 [Pre-pandemic]: (R) <20 years: *n =* 12 20–35 years: *n =* 230 >35 years: *n =* 37	No difference was found in depression and suicidal ideations in pregnant women in high-risk obstetric units during compared to before the pandemic	8
Silverman et al. (84)	Cross-sectional	United States of America	Obstetric clinics	**During social restrictions** (March 13–June 30, 2020; *n =* 252) vs. **Before social restrictions** (January 2–March 12, 2020; *n =* 264)	Postpartum women (*N =* 516)	R = 19–48 years	Postpartum women with low socio-economic status reported significantly fewer depressive symptoms after compared to before social restrictions were imposed.	6
Silverman et al. (85)	Cross-sectional	United States of America	Obstetric clinics	**During social restrictions** (May 4–June 12, 2020; *n*: NR) vs. **Before social restrictions** (February 2–March 11, 2020; *n*: NR)	Pregnant women receiving government-funded healthcare (i.e., low socio-economic status; *N =* 485)	R = 16–40 years	Pregnant women of low socio-economic status reported improved mood after compared to before social restrictions were imposed.	6
Sinaci et al. (86)	Cross-sectional	Turkey	High-risk pregnancy clinic	**Pandemic** (May–July 2020; *n =* 446) vs. **Pre-pandemic** (retrospective recall; *n =* 446)	Pregnant women (*N =* 446) • GA: 24.53 (M) years	Med = 28.93 R = 23.22–34.61 years	Participating pregnant women reported significantly higher trait anxiety during compared to before the pandemic. *Risk factor:* high-risk pregnancy	6
Suzuki (87)	Case-control	Japan	Postpartum outpatient clinic	**Pandemic** (March–April 2020; *n =* 132) vs. **Pre- pandemic** (March–April 2019; *n =* 148)	Postpartum women (*N =* 280)	R = <19 to >40 years	No difference was found in postpartum women's depressive symptoms before and during the pandemic. Postpartum women reported a decrease in mother-infant bonding during compared to before the pandemic.	7
Wu et al. (88)	Cross-sectional	China	Obstetric clinic	**After COVID-19 declaration** (January 20–February 9, 2020; *n =* 1,285) vs. **Before COVID-19 declaration** (January 1–20, 2020; *n =* 2,839)	Pregnant (3rd trimester) women (*N =* 4,124)	Med = 30 (R = 27–32) years	Pregnant women reported greater depression and self-harm after compared to before the COVID-19 declaration. *Risk factors (for depression):* increased information about COVID-19 and number of positive cases	6
Xie et al. (89)	Cross-sectional	China	Social media; Hospitals	**Pandemic** (January–August 2020; *n =* 689) vs. **Pre-pandemic** (March–December 2019; *n =* 2,657)	Pregnant women (*N =* 3,346) • GA: [pandemic] 16.10 (M) ± 5.0 (SD) weeks; [pre-pandemic] 16.24 (M) ± 5.0 (SD) weeks	[Pandemic]: 29.03 (M) ± 4.9 (SD) years [Pre-pandemic]: 28.94 (M) ± 6.4 (SD) years	Women pregnant during compared to before the pandemic reported greater depression, anxiety, and somatization, as well as lower family cohesion.	8
Zanardo et al. (90)	Case-control	Italy	Online	**Pandemic (**March 8–May 3, 2020; *n =* 91) vs. **Pre-pandemic** (March–May 2019; *n =* 101)	Postpartum women (*N =* 192) • GA: [pandemic] 39.41 (M) ± 1.12 (SD) weeks; [pre-pandemic] 39.42 (M) ± 1.14 (SD) weeks	[Pandemic]: 33.73 (M) ±5.01 (SD) years [Pre-pandemic]: 32.98 (M) ±5.07 (SD) years	Postpartum women reported higher depression during compared to before the pandemic.	7

*GA, gestational age; M, mean; SD, standard deviation; Med, median; R, range; PTSD, post-traumatic stress disorder; NR, not reported*.

a *This study covered children aged 0–8 years, but our data reviewed here only pertains to 0–18 months range*.

b *Assessed using a modified version of the Newcastle-Ottawa Scale (54). See section Assessment of Risk of Bias for details. Scores range from 0 (highest bias) to 9 points (lowest bias)*.

**Table 3:** Mental health outcomes in pregnant or postpartum women with vs. without COVID-19.

**Study**	**Study Design**	**Country**	**Study Period**	**Recruitment Sites/Methods**	**Participant Characteristics**			**Main Findings**	**Risk of Bias^a^**
					**Pregnancy/ Postpartum Status**	**Subgroups**	**Maternal Age**		
Bender et al. (91)	Cohort	United States of America	April 13-26, 2020 (during the pandemic)	Hospitals	Pregnant women (*N =* 318)	**COVID-19+** (*n =* 8) vs. **COVID-19–** (*n =* 310)	NR	Asymptomatic COVID-19+ pregnant women showed increased depression compared to asymptomatic COVID-19**–** pregnant women. This pattern extended to the early postpartum.	9
Ceulemans et al. (55)	Cross-sectional	United Kingdom, Norway, Switzerland, The Netherlands	June 16–July 14, 2020 (during the pandemic)	Online survey	Pregnant and postpartum women (*N =* 9,041)	**COVID-19+** (*n =* 56) vs. **COVID-19-** (*n =* 796)	NR	COVID-19+ pregnant and postpartum women were not more likely to have major depressive symptoms, generalized anxiety, or stress compared to COVID-19**–** women.	8
Kotabagi et al. (92)	Cross-sectional	United Kingdom	April 2020 (during the pandemic)	Hospitals	Pregnant women (*N =* 11) • GA: Med = 39 weeks	**COVID-19+** (*n =* 11)	Med = 31 years	COVID-19+ women reported an increase in psychological symptoms at the start of the pandemic, but symptoms decreased over time.	3

*GA, gestational age; M, mean; SD, standard deviation; Med, median; R, range; COVID19+, COVID-19 positive; COVID-19–, COVID-19 negative; NR, not reported*.

a *Assessed using a modified version of the Newcastle-Ottawa Scale (54). See section Assessment of Risk of Bias for details. Scores range from 0 (highest bias) to 10 points (lowest bias)*.

**Table 4:** Mental health outcomes in pregnant vs. non-pregnant women during the COVID-19 pandemic.

**Study**	**Study Design**	**Country**	**Study Period**	**Recruitment Sites/Methods**	**Participant Characteristics**			**Main Findings**	**Risk of Bias^a^**
					**Pregnancy/ Postpartum Status**	**Subgroups**	**Maternal Age**		
Lopez-Moralez et al. (93)	Longitudinal	Argentina	March 20–May 10, 2020 (during the pandemic)	Social media	Pregnant (GA: 20.05 [M] ± 8.70 [SD] weeks) and non-pregnant women (*N =* 204)	**Pregnant** (*n =* 102) vs. **Non-pregnant** (*n =* 102)	32.56 (M) ± 4.71 (SD) years	Compared to non-pregnant women, pregnant women showed increased depression, anxiety and decreased negative affect during the pandemic.	6
Yassa et al. (94)	Case-control	Turkey	April 2020 (during the pandemic)	Tertiary “coronavirus pandemic” hospital centre	Pregnant (GA: Med = 25 [R = 4–42] weeks) and non-pregnant women (*N =* 304)	**Pregnant** (*n =* 203) vs. **Non-pregnant** (*n =* 101)	[Pregnant]: 27.4 (M) ± 5.3 (SD) years [Non-pregnant]: 27.6 (M) ± 4.1 (SD) years	Compared to non-pregnant women, pregnant women reported lower anxiety and greater OCD-like symptoms during the pandemic.	7
Zhou et al. (95)	Cross-sectional	China	February 28–March 12, 2020 (during the pandemic)	Social media	Pregnant and non-pregnant women (*N =* 859)	**Pregnant** (*n =* 544) vs. **Non-pregnant** (*n =* 315)	[Pregnant]: 31.1 (M) ± 3.9 (SD) years [Non-pregnant]: 35.4 (M) ± 5.7 (SD) years	Compared to non-pregnant women, pregnant women reported low depression, anxiety, PTSD, and insomnia during the pandemic.	7

*GA, gestational age; M, mean; SD, standard deviation; Med, median; R, range; PTSD, post-traumatic stress disorder; OCD, obsessive-compulsive disorder*.

a *Assessed using a modified version of the Newcastle-Ottawa Scale (54). See section Assessment of Risk of Bias for details. Scores range from 0 (highest bias) to 10 points (lowest bias)*.

**Table 5:** Perinatal mental health outcomes during COVID-19 as a function of pregnancy-related factors (e.g., stage of pregnancy/postpartum, parity).

**Study**	**Study Design**	**Country**	**Study Period**	**Recruitment Sites/ Methods**	**Participants Characteristics**			**Main Findings**	**Risk of Bias^a^**
					**Pregnancy/ Postpartum Status**	**Subgroups**	**Maternal Age**		
Lebel et al. (96)	Cross-sectional	Canada	April 5–20, 2020 (during the pandemic); 2012–2016 (previous cohorts)	Social media	Pregnant women (*N =* 1,987)	**Nulliparous** (*n =* 971) vs. **Primiparous** (*n =* 735) vs. **Multiparous** (*n =* 277)	32.4 (M) ± 4.2 (SD) years	Regardless of parity, 37% of all participating pregnant women had elevated symptoms of depression and 56.6% had elevated levels of anxiety during the pandemic. Nulliparous, compared to primiparous or multiparous, women reported higher symptoms of pregnancy-related anxiety. *Protective factors:* social support, physical activity.	7
Saccone et al. (97)	Cross-sectional	Italy	March 15–April 1, 2020 (during the pandemic)	University	Pregnant women (*N =* 100)	**1st trimester** (*n =* 17) vs. **2nd trimester** (*n =* 35) vs. **3rd trimester** (*n =* 48)	NR	Pregnant women in the 1st trimester, compared to those in 2nd and 3rd trimesters, reported higher anxiety during the pandemic.	6
Shayganfard et al. (98)	Cross-sectional	Iran	Lockdown period (during the pandemic)	Hospital	Pregnant (GA: 27.20 [M] ± 5.77 [SD]; *n =* 66) and postpartum (<6 weeks; *n =* 37) women (*N =* 103)	**Before delivery** (*n =* 66) vs. **Post-delivery** (*n =* 37)	28.57 (M) ± 6.85 (SD) years	While women reported higher stress after compared to before delivery, no differences were found in depressive and anxiety symptoms between pregnant and postpartum women. *Risk factors (for anxiety and depression):* strict adherence to rules, worries and discomfort around post-poning/canceling routine medical care appointments, contact with/exposure to a person with COVID-19.	6
Stepowicz et al. (99)	Cross-sectional	Poland	April 7–May 24, 2020 (during the pandemic)	Hospital	Pregnant (*n =* 164) and postpartum (*n =* 46) women (*N =* 210)	**1st trimester** (*n =* 11) vs. **2nd trimester** (*n =* 46) vs. **3rd trimester** (*n =* 107) vs. **Postpartum** (*n =* 46)	Med = 31 (R = 19–45) years	Pregnant women in the 1st trimester, compared to women in the 2nd and 3rd trimesters or in the postpartum, reported higher levels of anxiety during the pandemic.	6
Wang et al. (100)	Longitudinal cohort	China	May 1–July 31, 2020 (during the pandemic)	National epidemic reporting system	Pregnant women (*N =* 138) • 1st trimester: *n =* 13) • 2nd trimester: *n =* 6 • 3rd trimester: *n =* 53	**Post-delivery** (*n =* 57) **vs. Post-abortion** (*n =* 15)	Med = 31 years	22% of participating women reported symptoms of depression and PTSD during the pandemic. There was no significant difference in symptoms between women who delivered vs. those who had induced abortion.	8
Zeng et al. (101)	Cross-sectional	China	March 25–June 5, 2020 (during the pandemic)	Hospital	Pregnant (3rd trimester; *n =* 516) and postpartum ( ≤ 1 week; *n =* 109) women (*N =* 625)	**3rd trimester** (*n =* 516) vs. **Postpartum** (*n =* 109)	29.2 (M) ± 4.2 (SD) years	Pregnant women in the third trimester were more likely to report depression and anxiety than postpartum women during the pandemic.	8
Zhang and Ma (102)	Cross-sectional	China	February–March 2020 (during the pandemic)	Social media	Pregnant women (*N =* 560)	**1st trimester** (*n =* 227) vs. **2nd trimester** (*n =* 220) vs. **3rd trimester** (*n =* 113)	25.8 (M) ± 2.7 (SD) years	Pregnant women paid significantly more attention to their mental health during the 3rd trimester, compared to first and second trimesters. *Risk factors*: increased stress from work/home, and helplessness/apprehension during the early stages of the pandemic.	7

*GA, gestational age; M, mean; SD, standard deviation; Med, median; R, range; PTSD, post-traumatic stress disorder*.

a *Assessed using a modified version of the Newcastle-Ottawa Scale (54). See section Assessment of Risk of Bias for details. Scores range from 0 (highest bias) to 10 points (lowest bias)*.

**Table 6:** Perinatal mental health outcomes during COVID-19 as a function of cultural or geographic factors.

**Study**	**Study Design**	**Country**	**Study Period**	**Recruitment Sites/Methods**	**Participant Characteristics**			**Main Findings**	**Risk of Bias^a^**
					**Pregnancy/ Postpartum Status**	**Subgroups**	**Maternal Age**		
Bo et al. (103)	Cross-sectional	China	February 22–March 10, 2020 (during the pandemic)	Social media	Pregnant and postpartum women (*N =* 1,309) • 1st & 2nd trimester: *n =* 373 • 3rd trimester: *n =* 545 • Postpartum: *n =* 391	**High-risk area** residents (Central/Western China; *n =* 418) vs. **Low-risk area** residents (Northern/Southern China; *n =* 891)	29.99 (M) ± 4.53 (SD) years	27.43% of participating women reported depression. Women living in high-risk area (central/western China), compared to women living in low-risk area (northern/southern China), were more likely to report depression. *Risk factors:* concerns about COVID-19 infection and delayed regular medical check-ups	6
Dong et al. (104)	Cross-sectional	China	February 22–27, 2020 (during the pandemic)	Social media and workplaces	Pregnant women (*N =* 156) • 0–12 weeks GA: *n =* 36 • 13–24 weeks GA: *n =* 46 • 25–40 weeks GA: *n =* 74	**High-risk area** residents (Wuhan; *n =* 101) vs. **Low-risk area** residents (other provinces in China; *n =* 55)	20–25 years: *n =* 4 26–30 years: *n =* 91 31–50 years: *n =* 61	There was no difference in depressive and anxiety symptoms in women living in high-risk area (Wuhan) compared to those living in other areas.	8
Liu et al. (17)	Cross-sectional	China	February 3–9 2020 (during the pandemic)	Hospitals	Pregnant women (*N =* 1,947) • 1st trimester: *n =* 83 • 2nd trimester: *n =* 639 • 3rd trimester: *n =* 1,125	**High-risk area** residents (Wuhan; *n =* 932) vs. **Low-risk area** residents (Chongqing; *n =* 1,015)	<35 years: *n =* 1,734 ≥35 years: *n =* 213	17.2% of participating women reported anxiety. Pregnant women residing in a high-risk area (Wuhan) reported higher anxiety compared to women residing in low-risk areas. (Chongqing)	9
Spinola et al. (105)	Cross-sectional	Italy	May 11–June 6, 2020 (lockdown period during the pandemic)	Social media	Postpartum women (<1 year; *N =* 243)	**High-risk area** residents (Northern Italy; *n =* 131) vs. **Low-risk area** (Central or Southern Italy; *n =* 109)	34.01 (M) ± 4.27 (SD) years	44% of participating women reported postpartum depression. Women who spent isolation in high-risk areas (northern Italy) reported greater postpartum depression and adopted more maladaptive coping strategies than women living in lower risk areas. *Risk factors:* prior abortion, previous psychiatric history, COVID-19 infection.	6
Taubman–Ben-Ari et al. (106)	Cross-sectional	Israel	March 18–28, 2020 (during the pandemic)	Social media	Pregnant women (*N =* 336) • GA: 25.42 (M) ± 9.57 (SD) weeks	**Ethnic minority** (Arab; *n =* 111) vs. **Ethnic majority** (Jewish; *n =* 225)	30.31 (M) ± 4.97 (SD) years	Women of ethnic minority (Arab) reported more anxiety symptoms than women of a majority ethnicity (Jewish)	7
Zhang et al. (107)	Cross-sectional	China	February 13–16, 2020 (during the pandemic)	Hospitals	Pregnant women (*N =* 1,901)	**High risk area** (Central China; *n =* 406) vs. **Low risk area** (Other provinces in China; *n =* 1,495)	28.9 (M) ± 4.7 (SD) years	Women living in the epicenter (Hubei) reported higher psychological symptoms, such as PTSD, during the pandemic than women in other provinces and in pre-pandemic samples.	6

*GA, gestational age; M, mean; SD, standard deviation; Med, median; R, range; PTSD, post-traumatic stress disorder*.

a *Assessed using a modified version of the Newcastle-Ottawa Scale (54). See section Assessment of Risk of Bias for details. Scores range from 0 (highest bias) to 10 points (lowest bias)*.

**Table 7:** Perinatal mental health outcomes during COVID-19 as a function of depression or anxiety severity.

**Study**	**Study Design**	**Country**	**Study Period**	**Recruitment Sites/Methods**	**Participant Characteristics**			**Main Findings**	**Risk of Bias^a^**
					**Pregnancy/ Postpartum Status**	**Key Variables Examined or Subgroups**	**Maternal Age**		
Dagklis et al. (108)	Cross-sectional	Greece	March 2020 (1st, 3rd, and 6th week of lockdown during the pandemic)	Prenatal clinic	Pregnant women (*N =* 269)	**State** and **trait anxiety** (State-Trait Anxiety Inventory)	≤ 35 years: *n =* 195 > 35 years: *n =* 74	Pregnant women reported higher state anxiety (i.e., anxiety during pregnancy) than trait-anxiety (i.e., lifetime anxiety) during lockdown. State anxiety fluctuated depending on the state of the pandemic and was positively associated with depressive symptoms. *Risk factors (for state anxiety):* early stages following lockdown, and third trimester of pregnancy	6
Durankuş & Aksu. (109)	Cross-sectional	Turkey	During the pandemic (specific dates: NR)	Online	Pregnant women (*N =* 260) • GA: 7.04 (M) ± 5.88 (SD) weeks	**High** (*n =* 92) vs. **Low** (*n =* 168) **depression** (Edinburgh Postnatal Depression Scale)	29.56 (M) ± 3.83 (SD) years	35.4% of participating women screened positive for depression. Pregnant women with high compared to low depressive symptoms reported more severe impact and social isolation during COVID-19 as well as higher anxiety symptoms. *Risk factors (for depression):* social isolation, greater number of children, and prior psychiatric history	7
Kahyaoglu & Kucukkaya (110)	Cross-sectional	Turkey	June–July 2020 (during the pandemic)	Social media	Pregnant women (*N =* 403) • GA: 27.3 (M) ± 8.8 (SD) weeks	**Anxiety** and **depression** (Hospital Anxiety and Depression Scale)	28.2 (M) ± 4.5 (SD) years	64.5% of participating pregnant women reported anxiety during COVID-19 and 56.3% reported depression. *Risk factors:* low education levels, low physical activity, discomfort during hospital/doctor's visits, lack of information around the effect of COVID-19 on pregnancy	6
Mappa et al. (111)	Cross-sectional	Italy	January–February 2020 (during the pandemic)	Prenatal clinics	Pregnant women (*N =* 178) • GA: Med = 18 weeks	**State** and **trait anxiety** (State-Trait Anxiety Inventory)	Med = 33 years	Pregnant women reported higher state anxiety during the pandemic than trait-anxiety (i.e., pre-existing anxiety). *Risk factor (for state anxiety):* high education status	7
Oskovi-Kaplan et al. (112)	Cross-sectional	Turkey	June 2020 (during the pandemic)	Obstetric tertiary care center with strong hospital restrictions	Postpartum women (<2 days; *N =* 223) • GA: Med = 39 weeks	**High** (*n =* 33) **vs. Low** (*n =* 190) **depression** (Edinburgh Postnatal Depression Scale)	Med = 26 years	14.7% of participating women screened positive for postpartum depression. Women who screened positive for postpartum depression, compared to those who did not, reported significantly lower mother-to-infant attachment.	6
Patabendige et al. (113)	Cross-sectional	Sri Lanka	April 27–May 20, 2020 (during the pandemic)	Prenatal clinics	Pregnant women (*N =* 257) • GA: 23.3 (M) ± 10.2 (SD) weeks	**Anxiety** and **depression** (Hospital Anxiety and Depression Scale)	29.2 (M) ± 5.7 (SD) years	17.5% of participating pregnant women reported anxiety and 19.5% reported depression. *Risk factors:* low maternal age (18–25 years), time spent watching television to seek COVID-19 information, and low household income	7
Ravaldi et al. (114)	Cross-sectional survey	Italy	March 18–31, 2020 (1st month of full lockdown during the pandemic)	Social media	Pregnant women (*N =* 737) • GA: Med = 27.8 (R = 4.7–42.5) weeks	**PTSD** (National Stressful Events Survey), **state** and **trait anxiety** (State and Trait Anxiety Inventory)	Med: 34.4 (R = 18.4–47.4) years	21.7% of participating women reported clinically significant anxiety and 10.2% reported clinically significant PTSD. Pregnant women with previous history of depression and/or anxiety reported elevated PTSD symptoms during the pandemic.	9
Sun et al. (115)	Cross-sectional	China	December 31, 2019–March 22, 2020 (during the pandemic)	Inpatient hospitals	Pregnant (>28 weeks; *n =* 738) and postpartum (<7 days; *n =* 2,092) women (*N =* 2,883)	**Depression** (*n =* 972) vs. **No depression** (*n =* 1,911) (Edinburgh Postnatal Depression Scale)	<25 years: *n =* 126 25–29 years: *n =* 1,194 30–34 years: *n =* 1,159 >34 years: *n =* 404	33.71% of the participating women had depression symptoms. Depressive symptoms increased among postpartum women as the pandemic worsened, which was then followed by a decrease in depressive symptoms among pregnant women as the pandemic became more under control. *Risk factors*: traumatic delivery, poor sleep quality, maternal/passive smoking, lack of exercise, and poor family functioning	6

*GA, gestational age; M, mean; SD, standard deviation; Med, median; R, range; PTSD, post-traumatic stress disorder; NR, not reported*.

a *Assessed using a modified version of the Newcastle-Ottawa Scale (54). See section Assessment of Risk of Bias for details. Scores range from 0 (highest bias) to 10 points (lowest bias)*.

**Table 8:** Perinatal mental health outcomes during COVID-19 as a function of factors not examined elsewhere.

**Study**	**Study Design**	**Country**	**Study Period**	**Recruitment Sites/Methods**	**Participant Characteristics**			**Main Findings**	**Risk of bias^a^**
					**Pregnancy/ Postpartum Status**	**Key Variables Examined Or Subgroups**	**Maternal Age**		
Ahorsu et al. (116)	Cross-sectional	Iran	March–April 2020 (during the pandemic)	Health and household registration system	Pregnant women (GA: 15.04 (M) ± 6.00 (SD) weeks; *n =* 290) and their husbands (*N =* 580)	**Fear of COVID-19**, COVID-19 preventive behaviors, depression, anxiety, mental quality of life	29.24 (M) ± 5.84 (SD) years	Pregnant women's own or partner's fear of COVID-19 was associated with increased depressive symptoms and lower mental health quality during the pandemic.	6
Chaves et al. (117)	Cross-sectional	Spain	April 7–May 8, 2020 (during the pandemic)	Social media	Pregnant (*n =* 450) and postpartum (<6 months; *n =* 274) women (*N =* 724)	**Life satisfaction**, depression, anxiety	33.36 (M) ± 4.12 (SD) years	58% of participating women reported depressive symptoms and 51% of women reported anxiety symptoms. *Risk factors (for life satisfaction):* [pregnancy] poor perceived self-health, single/separated marital status, health practitioner occupation status*;* [postpartum] poor perceived self-health, baby's poor health, poor quality of baby's sleep, single/separated marital status	6
Ding et al. (118)	Cross-sectional	China	March 7–23, 2020 (during the pandemic)	Hospitals	Pregnant women (*N =* 817) • 1st trimester: *n =* 115 • 2nd trimester: *n =* 247 • 3rd trimester: *n =* 455	**Knowledge, attitudes, and practices toward COVID-19**, anxiety	29.1 (M) ± 4.0 (SD) years	20.8% of participating pregnant women reported anxiety. *Risk factors:* low knowledge of the impact of COVID-19 on pregnancy, fear of COVID-19 infection, distrust in media, previous children in the family	7
Gildner et al. (119)	Cross-sectional	United States of America	April–June 2020 (during the pandemic)	Social media	Pregnant women (*N =* 1,856) • GA: 26.1 (M) ± 8.62 (SD) weeks	**COVID-related exercise change**, depression	31.3 (M) ± 4.30 (SD) years	Pregnant women reporting COVID-related changes in their exercise routine had higher depressive symptoms. Women living in metro compared to rural areas were more likely to report changes to exercise routine.	7
Harrison et al. (120)	Cross-sectional	United Kingdom	May 1–June 1, 2020 (during the pandemic)	Social media	Pregnant women (*N =* 205) • 1st trimester: *n* = 70 • 2nd trimester: *n* = 69 • 3rd trimester: *n* = 66	**Perceived social support**, depression, anxiety, repetitive negative thinking, loneliness	18–24 years: *n =* 13 25–34 years: *n =* 129 35–44 years: *n =* 63	Pregnant women experiencing low levels of perceived support reported more depressive and anxiety symptoms, which were mediated by increased repetitive negative thinking and loneliness.	6
Jiang et al. (121)	Cross-sectional	China	February 5–28, 2020 (during the pandemic)	Hospital	Pregnant women (*N =* 1,873) • 1st trimester: *n =* 598 • 2nd trimester: *n =* 703 • 3rd trimester: *n =* 572	**Access to prenatal care information**, depression, anxiety, perceived stress	29 (M) ± 4.10 (SD) years	45.9% of participating pregnant women reported depression, 18.1% reported anxiety, and 89.1% reported stress. Those who accessed prenatal care during the pandemic were at lower risk of perceived stress, anxiety, and depression	7
Kachi et al. (122)	Cross-sectional	Japan	May 22–31, 2020 (during the pandemic)	Workplaces	Pregnant women (*N =* 359) • 8–13 weeks: *n =* 39 • 14–27 weeks: *n =* 140 • 28–41 weeks: *n =* 180	**Maternity harassment** (pregnancy discrimination; *n =* 89) vs. **No maternity harassment** (*n =* 270)	[maternity harassment]: 31.3 (M) ± 4.8 (SD) years [no harassment]: 31.2 (M) ± 4.6 (SD) years	1/4 of pregnant women experienced maternity harassment in the workplace. Pregnant women who experienced maternity harassment had a 2.5-fold higher prevalence of depression than those who had not experienced harassment.	6
Lin et al. (123)	Online cross-sectional	China	February 17–March 16, 2020 (during the pandemic)	Obstetric clinics and hospitals	Pregnant women (*N =* 751) • 1st trimester: *n =* 514 • 2nd trimester: *n =* 214 • 3rd trimester: *n =* 23	**Sleep conditions during the pandemic**, depression, anxiety	30.51 (M) ± 4.28 (SD) years	35.4% of participating pregnant women reported anxiety and 13.4% reported depression. Pregnant women with poor sleep quality/duration were at higher risk of depressive and anxiety symptoms.	7
Shahid et al. (124)	Cross-sectional	Pakistan	August 6–20, 2020 (during the pandemic)	Outpatient department of obstetrics and gynecology hospital	Pregnant women (*N =* 552) • GA: 25.3 (M) ± 10.4 (SD) weeks	**Awareness and concerns about COVID-19**, depression, anxiety	32 (M) ± 7.3 (SD) years	64% of pregnant women reported a high level of awareness and concern about the COVID-19 pandemic, and were at high risk of depression and anxiety	7
Thayer & Gildner (125)	Cross-sectional	United States of America	April 16–30, 2020 (during the pandemic)	Social media	Pregnant women (*N =* 2,099) • GA: 26.4 (M) ± 9.0 (SD) weeks	**COVID-19 associated financial stress**, depression	31.3 (M) ± 4.4 (SD) years	43% of participating pregnant women experienced COVID-19 related financial stress and 24% had clinically significant depression. Those with high financial stress were at high risk of clinically significant depression.	6
Zhang et al. (107)	Retrospective	China	April 11–May 25, 2020 (during the pandemic)	Hospitals	Postpartum women (<1 week; *N =* 878)	**Emotional eating**, changes in dietary patterns	R: 18–45 years	Postpartum women during the pandemic reported a dietary change and higher emotional eating. *Risk factors (for emotional eating):* high-risk residence status (Southern China and Wuhan), low exercise, and high concern about COVID-19	6

*GA, gestational age; M, mean; SD, standard deviation; Med, median; R, range*.

a *Assessed using a modified version of the Newcastle-Ottawa Scale (54). See section Assessment of Risk of Bias for details. Scores range from 0 (highest bias) to 10 points (lowest bias)*.

**Table 9:** Modeling or intervention studies of perinatal mental health outcomes during the COVID-19 pandemic.

**Study**	**Study Design**	**Country**	**Study Period**	**Recruitment Sites/Methods from**	**Participant Characteristics**			**Main Findings**	**Risk of Bias^a^**
					**Pregnancy/ Postpartum Status**	**Key Variables Examined or Subgroups**	**Maternal Age**		
Aksoy Derya et al. (126)	Quasi-experimental	Turkey	April 22–May 13, 2020 (during the pandemic)	Prenatal classes	Pregnant women (*N =* 96) • GA: [intervention] 31.47 (M) ± 3.92 (SD) weeks; [control] 31.12 (M) ± 4.16 (SD) weeks	**Tele-education intervention** (for pregnancy and birth planning; *n =* 48) vs. **No intervention** (*n =* 48)	[intervention]: 28.70 (M) ± 4.73 (SD) years [control]: 28.06 (M) ± 4.12 (SD) years	Pregnant women who received the tele-education intervention reported less pregnancy-related anxiety than pregnant women who received no intervention.	6
Gamache et al. (127)	Single time point correlational	Canada	April 2–13, 2020 (during the pandemic)	Social media	Pregnant women (*N =* 1,207)	**Personality pathology**, affective/ behavioral/ thought problems, **mentalization of trauma**	29.6 (M) ± 4.0 (SD) years	Level of personality functioning had both direct and indirect (via mentalization of trauma) effects with affective/behavioral/thought problems in pregnant women during the pandemic.	6
Guo et al. (128)	Cross-validation	The Netherlands, China, Italy	[Netherlands] April 17–May 10, 2020; [Italy] April 21–June 13, 2020; [China] April 21–28, 2020 (during lockdown)	Social media, school and day care centers, research panels	Pregnant women from the Netherlands: *n* = 900; Italy: *n* = 641; China: *n* = 922	[Shared factors across cultures] **Pandemic-related stress**, **resilience** [Unique across cultures] grandparental support, father involvement, **family structure characteristics**	36.74 (M) ± 5.58 (SD) years	COVID-19-related stress and family conflict were risk factors and resilience was a protective factor for pregnant women's mental health in all three countries. *Risk factors unique to each country: (Netherlands)*: high maternal education and unemployment; *(Italy)*: maternal age and poor physical health; *(China)*: poor physical health, high socio-economic status, and low social support	6
Matsushima & Horiguchi (129)	Cross-sectional	Japan	May 31–June 6, 2020 (during the pandemic)	Companies providing services to pregnant & postpartum women	Pregnant women (*N =* 1,777) • 1st trimester: *n =* 235 • 2nd trimester: *n =* 741 • 3rd trimester: *n =* 801	Depression, anxiety, anhedonia, socio-demographic factors	<25 years: 5.35% 25–29 years: 29.21% 30–34 years: 37.20% 35+ years: 28.25%	17% of participating women screened positive for depressive symptoms. Risk for psychological symptoms increased with cancellation of planned informal support, increase in perceived risk for COVID-19 infection, household financial strain, and lack of social support. *Risk factors:* low maternal age, low wealth, unemployment, and no partner status.	7
Salehi et al. (130)	Cross-sectional	Iran	March–April 2020 (during the pandemic)	Hospital	Pregnant women (*N =* 222)	Fear of COVID-19, **anxiety of COVID-19**, depression, pregnancy-related concerns, **pregnancy-related happiness**	29.1 (M) ± 5.6 (SD) years	Pregnant women's mental health disorders during the pandemic were positively associated with anxiety of COVID-19 and negatively associated with happiness experienced during pregnancy.	7
Zheng et al. (131)	Cross-sectional	China	February 2020 (during the pandemic)	Hospital	Pregnant women (*N =* 331) • GA: R = 37–42 weeks	**Psychological response to the pandemic**, **security sense**, pregnancy stress	30.37 (M) ± 4.22 (SD) years	Fear and depressive symptoms were the most reported symptoms. Psychological response to the pandemic was positively associated with pregnancy stress, partially mediated by decreased security sense.	6

*GA, gestational age; M, mean; SD, standard deviation; Med, median; R, range*.

a *Assessed using a modified version of the Newcastle-Ottawa Scale (54). See section Assessment of Risk of Bias for details. Scores range from 0 (highest bias) to 10 points (lowest bias)*.

### Perinatal Mental Health Outcomes

We divide our results into studies reporting the following: (1) *prevalence* of mental health outcomes in pregnant or postpartum women during the COVID-19 pandemic (Table 1), (2) mental health outcomes in pregnant or postpartum women *before vs. during* the COVID-19 pandemic (Table 2), (3) mental health outcomes in pregnant or postpartum women *with vs. without* COVID-19 (Table 3), (4) mental health outcomes in *pregnant vs. non-pregnant* women during the COVID-19 pandemic (Table 4), (5) perinatal mental health outcomes during COVID-19 as a function of *pregnancy-related factors* (e.g., stages of pregnancy/postpartum or parity) (Table 5), (6) perinatal mental health outcomes during COVID-19 as a function of *cultural or geographical factors* (Table 6), (7) perinatal mental health outcomes during COVID-19 as a function of *depression or anxiety severity* (Table 7), (8) perinatal mental health outcomes during COVID-19 as a function of factors not examined elsewhere (Table 8), and (9) modeling or intervention studies of perinatal mental health outcomes during the COVID-19 pandemic (Table 9).

#### Prevalence of Mental Health Outcomes in Pregnant or Postpartum Women During COVID-19

Eighteen of the 81 studies reported on the prevalence of mental health outcomes in pregnant or postpartum women during the pandemic (Table 1). Sixteen studies reported on moderate to severe symptoms of depression and/or anxiety in pregnant or postpartum women during the pandemic, with prevalence rates ranging between 20 and 64% (55–70, 132). One study reported that around 14% of their sample of pregnant women had elevated anxiety during the pandemic, which was higher than that observed in pregnant or non-pregnant population prior to COVID-19 (71). In contrast, another study reported more than 1/3 of their sample of pregnant women had elevated depression and anxiety yet indicated that these were comparable prevalence rates to their pre-pandemic population (56).

#### Mental Health Outcomes in Pregnant or Postpartum Women Before vs. During COVID-19

Nineteen of the 81 studies compared mental health outcomes in pregnant or postpartum women before and during the pandemic (Table 2). Eleven of these studies reported elevated levels of clinically significant mental health symptoms such as depression and anxiety in pregnant or postpartum women during the COVID-19 pandemic (72–77, 81, 86, 88–90). Three studies specifically asked pregnant and postpartum women to report their symptoms during the pandemic as well as to retrospectively report their symptoms before the pandemic. These studies generally report higher levels of depression and anxiety during the pandemic (75), with one study showing that changes in pre- to post-COVID-19 pregnancy-related anxiety were associated with an increase in the number of COVID-19-related stressors (81) and another study documenting higher rates of trait anxiety specifically in the case of high-risk pregnancies (86).

Higher acute stress was documented in mothers delivering during compared to before the pandemic (78, 79), which was in turn related to increased symptoms of Post-Traumatic Stress Disorder (PTSD) and challenges with mother-infant bonding and breastfeeding (79). Additionally, trauma-related symptoms, including PTSD and dissociative symptoms, as well as thoughts of self-harm, were higher in pregnant women during compared to before the pandemic (73, 88). Postpartum mothers tended to report increased problems in bonding with their infant during compared to before the pandemic (87).

In contrast to studies that reported increased mental health symptoms during compared to before the pandemic, four studies (80, 82, 85, 87) reported decreased clinically significant depressive symptoms in pregnant women during the pandemic or shortly after the onset of the pandemic (80). An additional study (83) reported no difference in depressive symptoms between pregnant women before and during the COVID-19 pandemic. Two studies (84, 85) found that their sample of pregnant women had higher mood during compared to before the onset of COVID-19 community restrictions; a later study by the same research group found that pregnant women living in lower socioeconomic areas reported significantly fewer depressive symptoms *after* restrictions were imposed (84).

#### Mental Health Outcomes in Pregnant or Postpartum Women With vs. Without COVID-19

Three studies examined mental health outcomes in pregnant and postpartum women with confirmed positive COVID-19 diagnoses (55, 91, 92) (Table 3). Women who delivered during the pandemic with positive COVID-19 diagnosis had depression and anxiety that rose to a maximum at the height of the pandemic, and then decreased as more COVID-19-related information and guidelines were made public (92). Similarly, asymptomatic pregnant women with positive COVID-19 showed increased depression compared to those with negative COVID-19, a pattern which extended to the early postpartum (91). In contrast to these findings, one study suggested that pregnant and breastfeeding women with positive COVID-19 were not more likely to have major depressive symptoms, generalized anxiety, or heightened stress compared to those with negative COVID-19 (55).

#### Mental Health Outcomes in Pregnant vs. Non-pregnant Women During COVID-19

Three studies compared mental health outcomes in pregnant and non-pregnant women during the pandemic and report mixed findings (Table 4). One study documented a greater increase in depression, anxiety, and negative affect, coupled with a more pronounced decrease in positive affect, in pregnant compared to non-pregnant women during a 50-day quarantine period (93). However, another study found that pregnant women, despite displaying increased OCD symptoms, showed decreased overall anxiety compared to non-pregnant women (94). Another report suggested decreased symptoms of depression, anxiety, and PTSD in pregnant compared to non-pregnant women during the pandemic (95).

#### Perinatal Mental Health Outcomes During COVID-19 as a Function of Pregnancy-Related Factors (e.g., Stages of Pregnancy/Postpartum, Parity)

Seven of the 81 studies examined the role of pregnancy-related factors, such as trimesters or parity, in perinatal mental health outcomes during COVID-19 (Table 5). Four studies (97, 99, 101, 102) compared pregnant women in different trimesters during the pandemic. Women in the first trimester of pregnancy had higher anxiety and depression (101) and experienced more severe psychological impact (97) during the pandemic compared to women in the second or third trimesters or the postpartum period (99). On the other hand, women in the third trimester paid more attention to their mental health compared to those in first and second trimesters of their pregnancy (102). A study comparing pregnant and postpartum women found no differences in depressive and anxiety symptoms during pregnancy and the postpartum despite increased levels of stress associated with the postpartum period (98). Similarly, a study comparing women who delivered vs. had induced abortion during the pandemic also reported no between-group differences in depression in their sample (100). First-time pregnancy status emerged as a significant factor influencing perinatal mental health outcomes during the pandemic, with nulliparous women showing higher pregnancy-related anxiety compared to primiparous or multiparous women (96).

#### Perinatal Mental Health Outcomes During COVID-19 as a Function of Cultural or Geographic Factors

Six studies investigated the impact of the pandemic on perinatal mental health outcomes in the context of cultural or geographical factors (Table 6). In terms of cultural and ethnic differences, one study (106) examined pregnant Jewish and Arab women during the pandemic and reported that while general anxiety levels were quite high among both Arab and Jewish women, Arab women (ethnic minority) displayed higher COVID-19-related anxieties than Jewish women (ethnic majority). Regarding geographical differences, three studies compared pregnant women living in the epicenter of the pandemic (e.g., city of Wuhan or the province of Hubei) compared to those living in regions that were low risk for COVID-19 (17, 104, 133). Although higher rates of anxiety (17) and PTSD (133) were generally documented in pregnant women living in high-risk compared to low-risk regions, one study (104) found no difference in rates of depression or anxiety among pregnant women living in the epicenter vs. low-risk regions, which was attributed to transparent communication of information and increased social support. Similar patterns have been observed in postpartum women, with those isolating in high-risk areas of Italy reporting heightened perceived risk and greater postpartum depression compared to those isolating in low-risk areas (105).

#### Perinatal Mental Health Outcomes During COVID-19 as a Function of Depression/Anxiety Severity

Eight studies examining pregnant and postpartum women during the pandemic compared high vs. low depression scores (109, 112), depression vs. anxiety scores (110, 113), state vs. trait anxiety scores (108, 111), or the presence of depression vs. no depression (115) (Table 7). Pregnant women with higher depression displayed significantly higher anxiety (109) and lower mother-to-infant attachment (112). While some groups reported high rates of depression and anxiety in pregnant women (110, 113), others reported that depressive symptoms increased among postpartum women as the pandemic worsened, which was then followed by a decrease in depressive symptoms among pregnant women as the pandemic became more under control (115). One study indicated that pregnant women with a previous history of depression or anxiety had a significantly higher level of anxiety and PTSD symptoms during the pandemic (114), while two studies reported higher state-anxiety specific to the pandemic, compared to trait anxiety in pregnant women (108, 111).

#### Perinatal Mental Health Outcomes During COVID-19 as a Function of Factors Not Examined Elsewhere

Eleven studies examined additional factors (i.e., factors not examined elsewhere) that may influence perinatal mental health outcomes during COVID-19, such as emotional eating (107), exercise (119), sleep deprivation (123), financial stress (125), access to prenatal care (121), knowledge, concerns, or worries about COVID-19 (118, 124), social or marital support (116), and pregnancy discrimination (122) (Table 8). Pregnant women reporting changes in their exercise routine [encompassing both increase or decrease in exercise; (119)], decreased sleep (123), or increased COVID-19-related financial stress (125) were at greater risk for experiencing depression during the pandemic, while access to prenatal care information was associated with lower risk of perceived stress, anxiety, and depression (121). Concerns about COVID-19 also emerged as a significant factor affecting perinatal mental health, showing links to increased anxiety, depression, and sleep disturbance during pregnancy and the postpartum (117, 124), as well as high emotional eating in the postpartum (107). At the same time, knowledge of the impact of the pandemic on pregnancy was shown to serve as a protective factor for prenatal anxiety (118). Perceived social and marital support was also an important protective factor for perinatal depression and anxiety symptoms (120). Pregnant women's fear of COVID-19, depression, mental health, and COVID-19-related preventive behaviors were shown to be dyadically linked to their husbands' fear, mental health, and preventive behaviors (116). In contrast, maternity harassment (i.e., pregnancy discrimination) experienced in the workplace was linked to a 2.5-fold increased risk of depression in pregnant women during the pandemic (122).

#### Modeling or Intervention Studies of Perinatal Mental Health Outcomes During COVID-19

Five studies used various analytic approaches (e.g., structural equation modeling, path analysis) to examine mediating or moderating factors (127–131) associated with mental health outcomes in pregnant women, while one study used a quasi-experimental design (126) to study the effect of an intervention pregnant women received during the pandemic (Table 9). The following factors demonstrated positive associations with mental health disorders in pregnant women: anxiety and concerns related to COVID-19 (130), perceived risk for COVID-19 infection, financial difficulties, and low social support (129). In a study comparing risk and protective factors across three different countries, family conflict and pandemic-related stress emerged as important risk factors that were shared across China, Italy, and the Netherlands, whereas risk factors unique to each country involved variables associated with family structure or characteristics (128). Mediating variables identified are also of note. One study highlighted that lowered sense of security partially mediated the relationship between pregnant women's psychological response to the pandemic and their pregnancy stress (131). Another study demonstrated that failed mentalization of trauma partially mediated the association between personality pathology and affective, behavioral, and thought problems during the pandemic (127). An intervention study examined the effectiveness of tele-education classes for pregnancy and birth planning during the pandemic, which was shown to be associated with decreased prenatal distress and pregnancy-related anxiety (126).

### Risk and Protective Factors Associated With Perinatal Mental Health Outcomes

Risk and protective factors that emerged from the 81 perinatal mental health studies are summarized below.

#### Socio-Economic Factors

Increased depressive and anxiety symptoms during the pandemic were positively associated with the following socio-economic factors: low maternal age (68, 88, 113, 129), large hoursehold size (66, 109, 118), full-time employment status, high stress at work (81, 88, 91, 102, 129), and low income/financial strain (56, 73, 74, 94, 113, 125, 129). Regarding educational status, elevated symptoms during the pandemic were generally associated with fewer years of education in perinatal women and their partners (73, 74, 81, 109, 110); however, some studies reported a positive association with higher anxiety and more years of education (58, 111).

#### Psychological and Social Factors

The following psychological and social factors were positively associated with increased psychological symptoms: single mother status (129), prior history of traumatic birth or abortion (105, 115), psychiatric history prior to the pandemic (73, 74, 81, 105, 109, 114), recent stressful experiences in the past month (55) and history of abuse (68).

Perceived low social support and social isolation were associated with increased psychological symptoms (57, 61, 62, 65, 78, 96, 109, 115, 120, 129), and increased social support acted as a protective factor (71, 96). In addition, general conflict, poor family functioning and martial distress emerged as significant risk factors for perinatal anxiety during the pandemic (56, 72, 74, 81, 89, 98, 116, 117, 128). Pregnant women's own health anxiety (81, 98, 103, 117, 118, 129) and partner's perceived fear of COVID-19 (116) were understood to be contributing to pregnant women's increased anxiety and preventative behavior such as increased hygiene practices and social distancing (62, 71, 116, 134). Women strictly adhering to the rules and safety recommendations reported greater symptoms (98).

#### Physical and Obstetric Factors

Pregnant women with placental abnormality (88), pregnancy complication (60), high-risk pregnancy (68), and COVID-19 related symptoms (60, 105) were at risk of increased psychological symptoms. Increased psychological symptoms in the postpartum were associated with perceived poor health (their own and their baby) (117) and loss of childcare (81). Perinatal women who were obese (72) or underweight in the third trimester were more likely to experience increased depressive symptoms (88, 101). Good sleep, both in quality and duration (96, 115, 123), and exercise emerged as protective factors associated with low depressive and anxiety symptoms (75, 78, 110, 119).

#### Information Available on COVID-19 and Healthcare Support During the Pandemic

Updated knowledge of COVID-19 (such as knowledge of the number of cases increasing daily) appeared to increase the prevalence of depressive symptoms early in the pandemic (88, 113), and decrease depressive symptoms over time as more information became available to the general public (42) and as confirmed cases began to decrease (115). Similarly, when following perinatal women with COVID-19 over a 11-week period during the pandemic, anxiety levels peaked at the start of the outbreak and decreased as more information was published and shared with the general public about the virus (92). Women who did not have information about the effect of COVID-19 on pregancy were at higher risk of anxiety (110). Delay in or cancellation of planned informal support and reduced prenatal care were associated with an increase in depressive and anxiety symptoms (57, 81, 96, 98, 103, 129). Women who accessed prenatal care information (121) and perceived strong support from healthcare staff (67) were at significantly lower risk of psychological distress. In one study, pregnant women receiving a week of tele-education (interactive education booklets, and consultation via phone calls) were significantly less likely to present with prenatal distress and anxiety compared to a control group of pregnant women who received no intervention (126).

### Risk of Bias

Scores from risk of bias and quality assessment are listed in Tables 1–9. The scores range from 3 (a moderate risk of bias) to 9 (a very low risk of bias), with the majority of studies ranging between risk of bias scores of 6–7, which are considered to present moderately to sufficiently robust and valid data. Methodological limitations identified as contributing to potential sources of bias included: (a) comparisons of the prevalence of mental health symptoms before and during the pandemic without using an interrupted time series analysis to minimize bias, (b) data collection method involving retrospective recall of symptoms prior to the pandemic, which is susceptible to recall bias, (c) convenience sampling such as online recruitment of participants, which served as a safe and effective data collection strategy during the pandemic but may have made it difficult to obtain a truly representative sample, and (d) small sample sizes given both the challenges and urgency of obtaining data during the pandemic.

## Discussion

We have conducted a systematic review summarizing both obstetric and mental health outcomes in perinatal women during the COVID-19 pandemic. At the time of our search, there is still limited data on the impact of COVID-19 on perinatal mental health, and obstetrics guidance continues to be updated as new evidence emerges. Perinatal mental health studies reviewed here overall converge to point to higher rates of depressive and anxiety symptoms in perinatal women during the pandemic compared to perinatal women prior to the pandemic. Financial strain, decreased social and family support, and low education emerged as key sociodemographic factors associated with increased depression and anxiety in perinatal women, while adequate sleep, moderate physical activity, and positive social support were negatively associated with these symptoms amidst the pandemic.

### Synthesis of Obstetric and Perinatal Mental Health Data

We propose that the degree to which COVID-19 increases mental health risk in perinatal women may be closely associated with their perception of risks present during the pandemic, their perceived sense of control in relation to the risk, their manner of coping in response to day-to-day stressors posed by the pandemic, and the level of support they have in their environment. Here we examine these factors in the context of the obstetric and neonatal data reviewed above to help make sense of the perinatal mental health data that have been reported during the first year of the COVID-19 pandemic.

### Uncertainty, Unpredictability, and Perceived Lack of Control

The data reviewed above suggest that perinatal women have been faced with a heightened sense of uncertainty and unpredictability during the pandemic around the possibility of contracting COVID-19, as well as constantly evolving estimates of the risk of COVID-19 to their pregnancy and newborn. Changing obstetric and public health guidelines, which could substantially change how they will receive perinatal care, deliver their baby, and care for their baby in the postpartum, likely further contributed to a lack of sense of predictability and control. Perceived lack of sense of control over one's environment has long been known to exacerbate increased distress in the face of a potential threat (135) and has been linked to increased depression and anxiety in both non-perinatal and perinatal populations (136). Our findings are in line with this, demonstrating a spike in perinatal anxiety and depressive symptoms in the face of a novel virus and an eventual decrease in symptoms as more information and guidelines became available.

### Adjustment Stress

The data reviewed above further suggest that COVID-19 increased the magnitude of stress with which perinatal women must cope during their transition to motherhood. The transition to motherhood can be characterized as a major physiological, psychological, and social adjustment for the mother. Successful adjustment to these changes is key to healthy perinatal adaptation, whereas adjustment difficulties contribute to perinatal depression and anxiety (137–139). During COVID-19, perinatal women have additionally had to contend with significant changes in the public's behaviors (e.g., stockpiling and quarantining), constantly evolving public health guidelines, and rapid changes in their expectations and plans about their pregnancy, while, in many cases, also managing heightened anxiety and stress of others (e.g., family members).

### Lack of Social Support

Restrictions that have been put in place to mitigate the risk of COVID-19, such as quarantine, physical distancing, and telehealth appointments, appear to have had the effect of undermining practical and emotional support provided to perinatal women, which is critical in reducing the risk of depression and anxiety in new mothers (140). As was discussed above, social isolation (96, 109) and decreased social support, as well as increased tension in marital or family relationships (74, 96, 115), were found to negatively impact perinatal mental health during COVID-19. In addition, the increased demands of home life during the pandemic (caring for other children, homeschooling or remote work), complicated by reduced support available from family members or paid childcare, may further interfere with the mother's ability to meaningfully connect with her baby (e.g., skin-to-skin contact and breastfeeding), which typically contribute to the mother's sense of joy and reward despite the stress of transition.

### Protecting Perinatal Mental Health

Efforts should be focused on reducing the magnitude of stress and a perceived sense of lack of control experienced by perinatal women, while increasing their capacity for coping and level of social support, as well as promoting adequate sleep and exercise. Considering the findings demonstrating that distress around COVID-19 was linked dyadically between perinatal women and their partners, preventative measures should involve partners as well as perinatal women. Empirically based interventions that help examine one's capacity for control and enhance realistic appraisal of potential risks, while targeting stress reduction and enhanced coping will be helpful for perinatal women experiencing increased distress during COVID-19. Mindfulness, distress tolerance skills, relaxation exercises, and interpersonal relationship skills can be helpful skills-based exercise for this population.

### Respectful Care During Pregnancy and Postpartum

It is of paramount importance to consider the fundamental human rights of pregnant and postpartum women and their newborns when accessing health services related to pregnancy, childbirth, and postpartum care. Comprehensive guidelines and frameworks have been created by national and international organizations such as the RCOG (141) and the WHO (142) to ensure evidence-based intrapartum care, regardless of the setting or level of health care. Perinatal experts have highlighted the need for maintaining respectful care for all women and newborns, especially during the pandemic (143–145), to reduce the traumatic impact the pandemic may have on women and children. Respectful maternal and newborn care involves effectively communicating all available options to pregnant women (in a manner that is accessible to women of a wide range of language and literacy levels), allowing companion of choice to be present during childbirth for support and to advocate for pregnant women, as well as ensuring equal access to the same range, quality, and standard of care across any marginalized or underserved groups. We recognize respectful maternal and newborn care as having the potential to improve physical and mental health outcomes for perinatal women and their newborns. The quality of care provided to perinatal women during the pandemic may have influenced the mental health outcomes reported in this systematic review, and future studies will need to explore this in further detail.

### Strengths and Limitations

Our systematic review evaluates the impact of COVID-19 on mental health outcomes in pregnant and postpartum women, considering current obstetric and neonate guidelines. Limitations of the study include the following. First, many of the studies reviewed here include data from the early stage of the outbreak. As the COVID-19 pandemic progresses, evolving guidelines within the medical community and the larger society may continue to influence patterns of obstetric and perinatal mental health outcomes. Second, as with all traumatic events, the pandemic will have both acute and delayed effects on mental health. Our current review concerns the first year of the pandemic and underscores its acute effects. However, we are yet to learn about its longer-term impact on perinatal mental health during the second year of the pandemic and beyond, which would be critical to study and may vary from the effects reviewed here. Third, despite the global nature of the COVID-19 pandemic, it is important to note that different countries have been impacted differently by the pandemic, especially as new variants are introduced, and vaccination efforts unfold at different pace around the world. Fourth, variability in how perinatal mental health outcomes were measured and reported limited our ability to directly compare and synthesize outcomes across studies. Finally, as we have noted above with regard to our risk of bias assessment, a small proportion of studies reviewed here were identified as having a high risk of bias, primarily due to small sample size or the use of retrospective recall of mental health symptoms. In our efforts to provide a comprehensive picture of perinatal mental health studies from the first year of the pandemic, we chose not to exclude any studies on the basis of the risk of bias scores but have included the risk of bias score in the Tables for readers' review.

### Future Directions

As we enter a new chapter of the COVID-19 pandemic with the onset of new virus variants and the development of vaccines, large-scale studies and surveillance data that track the trajectory of perinatal mental health outcomes will continue to provide a more comprehensive picture of the impact of COVID-19 (146). Careful attention should be paid to methodological issues identified in the quality assessment described above (see section Risk of Bias). The COVID-19 pandemic has uncovered several inequalities and the urgent need to identify and prioritize high-risk groups of women who are more significantly and disproportionately impacted by the virus. Future studies should consider the variability present in a range of risk factors (including health disparities) and application of the framework and guidelines of respectful maternal and newborn care in measuring obstetric, neonatal, and perinatal mental health outcomes. During this second year of the pandemic, it would also be crucial that studies examine how the pandemic may have disparate effects among women and newborns in different parts of the world.

### Conclusion

This systematic review examined the impact of COVID-19 on mental health outcomes in pregnant and postpartum women in the context of obstetric and neonatal data that have thus far emerged. We have underscored risk and protective factors associated with perinatal mental health. There is an evident gap in our understanding of the long-term impact of outcomes reported here, and it is imperative that further attention and research be focused on the perinatal period, arguably the most significant and sensitive time of a mother and child's life, in the face of ongoing crisis.

## Author Contributions

UI, BJ, and HH conducted the systematic review, including the literature search, and analysis. UI, BJ, HH, and SK wrote the draft of the paper. SK added clinical and critical insight to the overall paper structure. All authors contributed to the article and approved the submitted version.

## Conflict of Interest

The authors declare that the research was conducted in the absence of any commercial or financial relationships that could be construed as a potential conflict of interest.
