# Supplementary material for: One Year Into the Pandemic: A Systematic Review of Perinatal Mental Health Outcomes During COVID-19
Source: Front Psychiatry. 2021 Jun 24;12:674194. doi: 10.3389/fpsyt.2021.674194 (PMC8264436; doi:10.3389/fpsyt.2021.674194)
Supplement: Supplementary file 1 [file Table_1.docx]

SUPPLEMENTARY MATERIAL

**Appendix 1: Supplementary Material**

SEARCH STRATEGY

Search terms and combinations used for 3 databases: EMBASE, MEDLINE, PsycInfo,

| 1 | covid-19 |
| --- | --- |
| 2 | novel corona* |
| 3 | SARS 2 covid-19 |
| 4 | sars cov 2 |
| 5 | corona |
| 6 | coronavirus |
| 7 | pregnan* |
| 8 | perinatal |
| 9 | neo-natal |
| 10 | post-partum |
| 11 | post-natal |
| 12 | maternal* |
| 13 | mother* |
| 14 | depress* |
| 15 | anxi* |
| 16 | psych* |
| 17 | mental health |
| 18 | attachment |
| 19 | well-being |
| 20 | 1 OR 2 OR 3 OR 4 OR 5 OR 6 |
| 21 | 7 OR 8 OR 9 OR 10 OR 11 OR 12 OR 13 |
| 22 | 14 OR 15 OR 16 OR17 OR 18 OR 19 |
| 23 | 20 AND 21 AND 22 |
|  |  |
